# Estradiol-17β Injection Induces Ovulation in Llamas

**DOI:** 10.3389/fvets.2020.576204

**Published:** 2020-10-15

**Authors:** Carolina P. Bianchi, Micaela A. Benavente, Florencia Viviani, María F. Gallelli, Marcelo A. Aba

**Affiliations:** ^1^Laboratorio de Endocrinología, Facultad de Ciencias Veterinarias, Universidad Nacional del Centro de la Provincia de Buenos Aires (UNCPBA), Tandil, Argentina; ^2^Centro de Investigación Veterinaria Tandil, Consejo Nacional de Investigaciones Científicas y Técnicas de Argentina, Tandil, Argentina; ^3^Instituto de Investigación y Tecnología en Reproducción Animal, Facultad de Ciencias Veterinarias, Universidad de Buenos Aires, Consejo Nacional de Investigaciones Científicas y Técnicas de Argentina, Tandil, Argentina

**Keywords:** estradiol-17β, ovulation, corpus luteum, progesterone, llamas

## Abstract

This study aimed to investigate the effect of three different doses of estradiol-17β on ovulation and subsequent luteal development and function in llamas. Twenty-three llamas were examined daily by transrectal ultrasonography until the detection of an ovulatory follicle (≥8 mm). Thereafter, animals were divided into five groups: Control (*n* = 3; treated with 1.6 ml of saline solution), GnRH group (*n* = 6, treated with an intravenous injection of 8.4 μg Buserelin), and estradiol groups that received 0.6 mg (E1, *n* = 4), 1 mg (E2, *n* = 4), or 1.6 mg (E3, *n* = 6) of estradiol-17β intravenously. Detection of ovulation was based on ultrasonographic visualization of disappearance of the largest follicle and subsequent presence of a newly formed corpus luteum (CL) and progesterone concentration exceeding 1 ng ml^−1^. Daily blood samples were collected to determine plasma progesterone concentration. Ovulation rate was 0% for control and E1 groups, 25% for E2 group, and 100% for GnRH and E3 groups. Differences in the mean CL diameter between GnRH and E3 groups were not statistically significant. Plasma progesterone concentration was similar between groups during the different days in ovulated animals. However, the day that the plasma progesterone concentration was above 1 ng ml^−1^ and the day that the highest plasma progesterone concentration was achieved differed among E3 and GnRH groups, occurring later in females treated with estradiol. In conclusion, an injection of estradiol-17β is capable of inducing ovulation in llamas and the response depends on the dose used. Most of the animals required the highest tested dose (1.6 mg) to induce the ovulatory process. Although the CL diameter in females induced to ovulate with estradiol was similar to that in llamas induced to ovulate with a GnRH analog, the rise in plasma progesterone concentration above 1 ng ml^−1^ and the peak progesterone concentration were attained 1 day later in the estradiol treated females.

## Introduction

Unlike other species such as sheep and cattle, which ovulate spontaneously as part of their estrous cycle, female camelids are induced ovulators requiring an external stimulus in the presence of a follicle with a diameter ≥ 7 mm to elicit the ovulatory process (1, 2). The luteinizing hormone (LH) concentration increases by 20 min after copulation, peaks within 2–3 h, remains high during 5 h, and reaches basal concentration by 12 h post-mating (3). An ovulation-inducing factor identified as beta nerve growth factor (4, 5) is present in the seminal plasma of males, which induces the LH secretion and ovulation around 30 h post-mating (6). Besides, the administration of exogenous hormones that elicit LH release such as gonadotrophin-releasing hormone (GnRH) or hormones with LH activity such as human chorionic gonadotrophin (hCG) induces ovulation within an interval of ~29 h [GnRH: (7)] or 26 h [hCG: (1)].

In spontaneously ovulating species, increasing plasma concentration of estradiol-17β, in the absence of plasma progesterone concentration from a corpus luteum (CL), induces the preovulatory surge of LH and, consequently, ovulation (8, 9). Moreover, treatment of heifers with estradiol-17β, without exogenous progesterone or progestogen, is followed by a surge release of LH (10, 11).

In llamas, the LH secretion and the ovulatory response depend on follicular size at the time of mating. Females with follicles ≥7 mm in diameter ovulate while those with smaller ovarian follicles (<6 mm) release less LH and do not ovulate in response to copulation suggesting that this pattern could be due to a reduced estrogen priming of the hypothalamus–pituitary axis (2). Plasma estradiol-17β concentration follows a wavelike pattern with basal concentration recorded, when small follicles are present in the ovaries (12). Although the increase in plasma estrogen concentrations does not elicit the LH preovulatory peak in llamas, it has been suggested that estradiol modulates the LH secretion by the pituitary in response to the intramuscular injection of purified ovulation-inducing factor in this species (13).

All these information show some evidence that estradiol modulates LH secretion in camelids; however, to our knowledge there are no reports evaluating the effect of exogenous administration of estrogens on the ovulatory process in these species. Thus, this study aimed to investigate the effect of three different doses of estradiol-17β on ovulation and subsequent luteal development and function in llamas. The hypothesis was that estradiol-17β is capable of triggering the ovulatory process in llamas and that the response depends on the dose used.

## Materials and Methods

Field studies were performed in compliance with animal welfare regulations set by the Faculty of Veterinary Sciences, UNCPBA, where activities were conducted. Animals belong to the Faculty of Veterinary Sciences, UNCPBA, and facilities are located in Tandil, Argentina, at 37°S, 60°W. Twenty-three nonpregnant, non-lactating female llamas, ranging between 5 and 12 years of age and with an average body weight of 100 ± 15 kg, were included in the study. All animals were clinically and reproductively healthy with a mean body condition score of 3 (body condition score from 1 = thin to 5 = obese) (14). Llamas were kept in pens isolated from males and fed pasture hay and water *ad libitum*.

### Experimental Design

Female llamas were examined daily by transrectal ultrasonography (Mindray, DP 6600 Vet, with 5.0/7.5 variable traducer probe) to assess ovarian status. When a growing follicle with a diameter ≥8 mm, considered ovulatory in this species (2), was observed, animals were randomly assigned to one of five treatments: control group (1.6 ml saline solution, im; *n* = 3); GnRH group (8.4 μg acetate of Buserelin, Receptal®, Intervet, Argentina, iv; *n* = 6) and E1, E2, and E3 groups that received 0.6 (*n* = 4), 1 (*n* = 4), or 1.6 mg (*n* = 6), respectively, of an intramuscular injection of estradiol-17β (17β-estradiol, Rio de Janeiro, Argentina) into the hind leg. Detection of ovulation was based on ultrasonographic visualization of disappearance of the largest follicle and subsequent presence of a newly formed CL and progesterone concentration exceeding 1 ng ml^−1^.

Thereafter, ultrasonographic examination was performed daily for 12 days to measure the CL diameter, which was estimated by averaging two measurements of the CL diameter at right angles to each other.

Blood samples were collected daily from the day that a growing follicle ≥8 mm was detected (Day 0) and during 12 days in ovulating animals. Blood samples were collected by jugular venipuncture in tubes with heparin (Heparin Sodium, SOBRIUS®, Fada Pharma, Buenos Aires, Argentina) and centrifuged immediately after collection. Plasma was stored at −20°C until hormone assays were performed.

### Hormone Determinations

Progesterone was measured using a RIA kit (IM 1188, Beckman Coulter, Immunotech, Czech Republic) previously validated for use with llama blood plasma (15). The intra-assay coefficient of variation was below 5% for concentrations between 0.1 and 40 ng ml^−1^. The inter-assay coefficients of variation for three quality-control samples were 2.9% (0.5 ng ml^−1^), 2.2% (2.0 ng ml^−1^), and 5.8% (10 ng ml^−1^). The sensitivity of the assay was 0.10 ng ml^−1^.

### Statistical Analysis

Follicle diameters at treatment and at Day 2 were compared among groups by one-way ANOVA followed by Tukey's test.

Normal distribution of the variables was tested in all cases using the Shapiro–Wilk test. For non-normally distributed variables (first day that a CL was detected, day of maximum CL diameter, first day plasma progesterone concentration was above 1 ng ml^−1^, and day that plasma progesterone concentration returned to values below 1 ng ml^−1^) the Mann–Whitney test was used to compare GnRH and E3 groups. For the remaining characteristics (maximum CL diameter, maximum progesterone concentration and day of maximum progesterone concentration), a Student *t*-test was applied.

The diameter of the CL and plasma progesterone concentration on the different days and between GnRH and E3 groups were analyzed by repeated measures one-way ANOVA or one-way ANOVA, respectively, followed by Tukey's test.

The percentage of animals that ovulated after the different treatments was compared using Fisher's exact test.

All statistical analyses were conducted using the Infostat Professional 2017 software package. Data are presented as mean ± S.E.M. Differences were considered to be significant when *P* < 0.05, and a tendency was considered when *P* < 0.1.

## Results

Mean diameter of the largest follicle at the time of treatment was similar among groups (*P* = 0.39). Ovulation rate differed between groups (*P* = 0.0001). All females treated with 1.6 mg of estradiol-17β (E3 group) ovulated after treatment as all animals injected with Buserelin and only one llama treated with 1 mg of estradiol-17β (E2 group). Ovulation, assessed by the disappearance of the largest follicle, was observed 48 h post injection (Day 2) in all llamas that ovulated in response to the different treatments. Mean diameter of the largest follicle on Day 2 differed among groups (*P* = 0.0001) (Table 1).

**Table 1:** Effect of the different treatments on ovulation in llamas (mean ± SEM).

	**Control**	**GnRH**	**E1**	**E2**	**E3**
Mean follicular diameter (mm) on day 0	8.2 ± 0.62^a^	9.7 ± 0.81^a^	9.5 ± 0.47^a^	9.6 ± 0.36^a^	10.0 ± 0.31^a^
Ovulation rate after treatment (%)	0 (0/3)^a^	100 (6/6)^b^	0 (0/4)^a^	25 (1/4)^a^	100 (6/6)^b^
Mean follicular diameter (mm) on Day 2	9.7 ± 0.68^x^	3.0 ± 0.42^y^	11.4 ± 1.44^x^	8.1 ± 2.02^x^	2.8 ± 0.43^y^

*Values with different superscripts within a row are significantly different*.

*a vs. b = Proportions with different superscripts are different (P = 0.0001)*.

*x vs. y = differences in mean follicular diameter on Day 2 between groups (P = 0.0001)*.

A CL was first detected after treatment between Days 4 and 5 in GnRH and E3 groups and on Day 4 in the only llama that ovulated in the E2 group. Maximum CL diameter (*P* = 0.73) and the day on which maximal CL diameter was observed (*P* = 0.70) were not different among groups (Table 2).

**Table 2:** Effect of administration of GnRH and 1.6 mg of estradiol-17β (E3 group) on luteal development in llamas (mean ± SEM).

	**GnRH**	**E3**
First day CL detected (day)	4.2 ± 0.31^a^	4.5 ± 0.22^a^
Maximum CL diameter (mm)	14.8 ± 1.14^a^	14.3 ± 0.85^a^
Day maximum CL diameter (day)	7.8 ± 0.31^a^	8.2 ± 0.31^a^
First day progesterone concentration > 1 ng ml^−1^ (day)	4.5 ± 0.22^a^	5.2 ± 0.20^b^
Maximum progesterone concentration (ng ml^−1^)	7.1 ± 1.29^a^	6.8 ± 1.73^a^
Day maximum progesterone concentration (day)	7.0 ± 0.37^a^	8.8 ± 0.49^c^
Day progesterone concentration <1 ng ml^−1^ (day)	10.0 ± 0.52^a^	11.4 ± 0.40^d^

*Values with different superscripts within a row are significantly different (a vs. c P = 0.015) or show a tendency to be different (a vs. b P = 0.07 and a vs. d P = 0.09)*.

**Figure 1:**
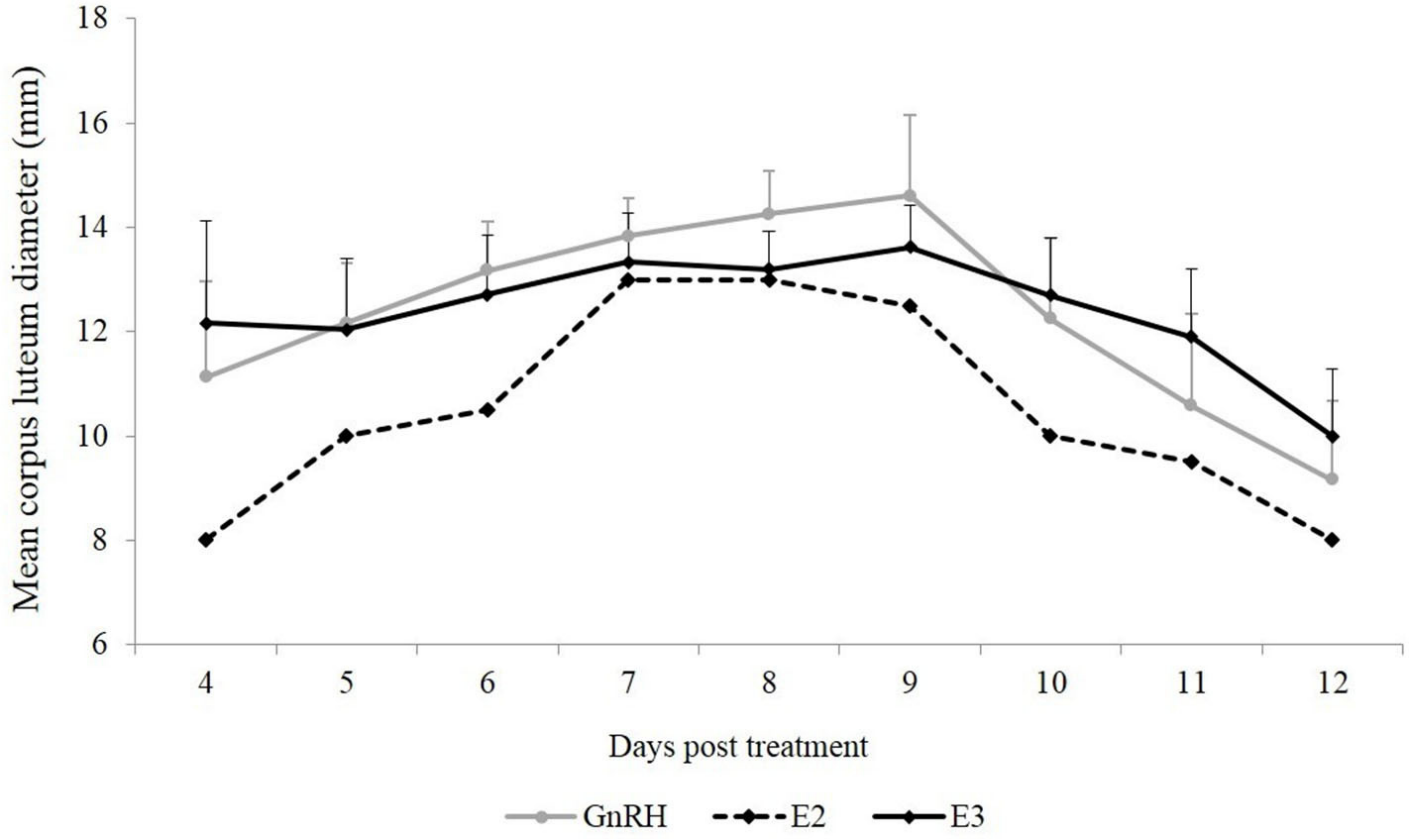
Mean CL diameter (mm) from the first day that CL was detected until Day 12 post-treatment. Gray line: animals treated with Buserelin, analog of GnRH (*n* = 6), black line: animals treated with a dose of 1.6 mg of estradiol-17β (*n* = 6), and dotted line represents the only llama that ovulated in response to a dose of 1.0 mg of estradiol-17β.

Mean CL diameter was similar between both groups during the experimental period (*P* = 0.90). An effect of day was observed in the GnRH and E3 groups (*P* < 0.01). Mean CL diameter in the GnRH group was smaller on Days 11 and 12 than on Days 6, 7, 8, and 9, and mean CL diameter in the E3 group was smaller on Day 12 than on Days 7, 8, and 9 (Figure 1).

The first day that the plasma progesterone concentration was above 1 ng ml^−1^ tended to be different between GnRH and E3 groups, occurring earlier in the first group (*P* = 0.07). The highest plasma progesterone concentration did not differ between both groups (*P* = 0.88). However, the day that the highest plasma progesterone concentration was achieved differed and occurred between Days 6 and 8 in the GnRH group and between Days 7 and 10 in the E3 group (*P* = 0.015). The highest plasma progesterone concentration in the llama that ovulated in the E2 group was recorded on Day 8 post treatment. The day that basal plasma progesterone concentration (below 1 ng ml^−1^) was observed tended to be different between both groups (*P* = 0.09) and was earlier in the GnRH group (Table 2).

**Figure 2:**
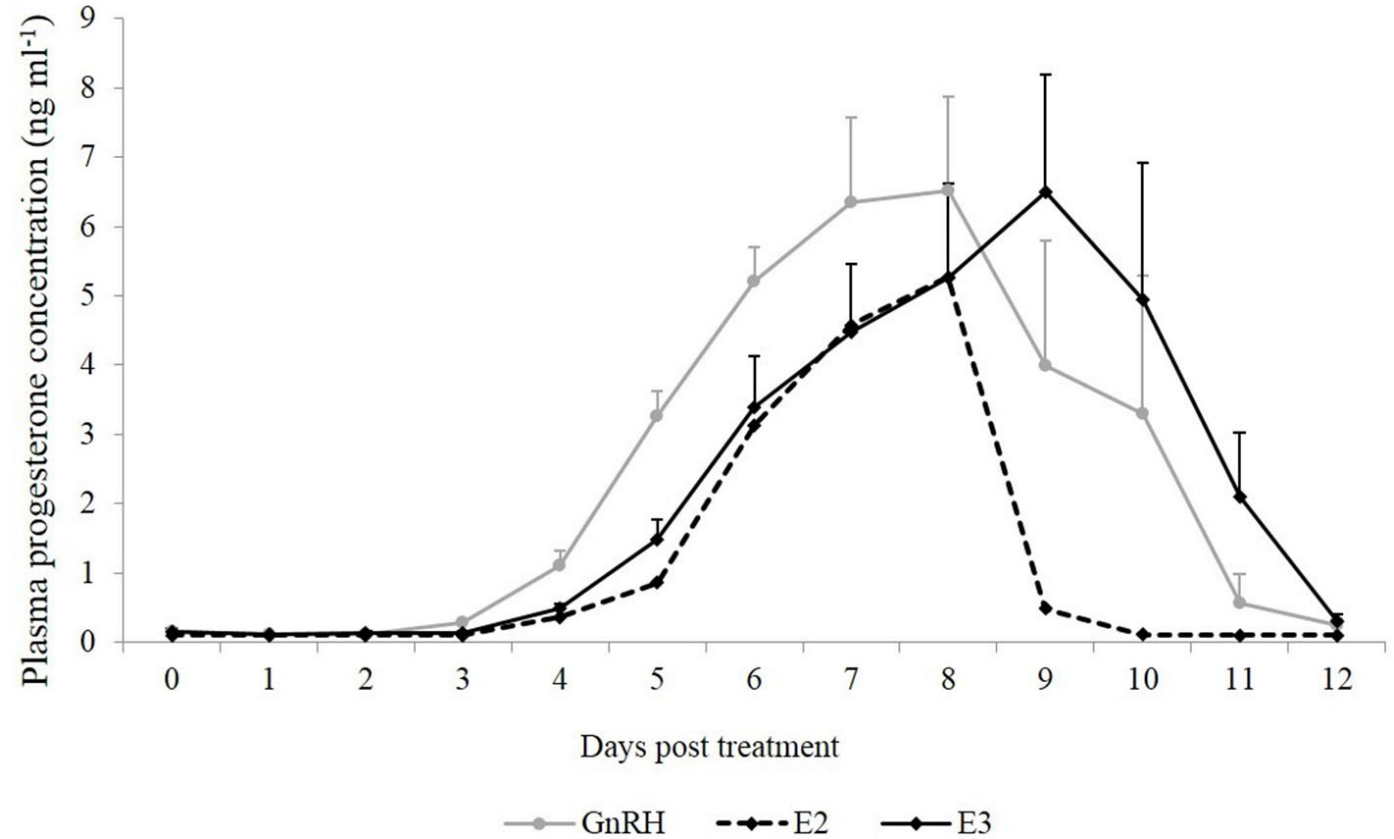
Mean plasma progesterone concentration (ng ml^−1^) from the day of treatment (Day 0) until Day 12 post-treatment. Gray line: animals treated with Buserelin, analog of GnRH (*n* = 6), black line: animals treated with a dose of 1.6 mg of estradiol-17β (*n* = 6), and dotted line represents the only llama that ovulated in response to a dose of 1.0 mg of estradiol-17β.

Plasma progesterone concentration was similar between groups during the different days (*P* = 0.68). An effect of day was observed in both groups (*P* < 0.0001), and the highest plasma progesterone concentration was reached on Days 7, 8, and 9 post treatment (Figure 2).

## Discussion

To our knowledge, this is the first report demonstrating that an injection of estradiol-17β is effective to induce ovulation in llamas being the response dependent of the dose. Previously, the administration of estradiol-17β was used in an attempt to control ovarian follicular development in alpacas. D'Occhio et al. (32) have reported that doses of 0.5 or 2 mg of estradiol-17β induce follicular regression and a new wave emergence regardless of the stage of follicular development without reporting ovulations. In a later study, using a dose of 1 mg of estradiol-17β, the author has observed that the diameter of the largest follicle remained >6 mm during all the study, indicating that ovulation did not occur (16). The disclosure with the later study could be related to the dose used. In the present study, an effect of the dose of estradiol-17β was demonstrated, observing ovulation in 100% of the animals when the highest dose (1.6 mg) was tested. Similarly, a positive correlation between the dose of estradiol and the magnitude of the LH surge was reported in other species such as cows (17, 18) and ewes (19, 20). Although llamas are induced ovulators requiring copulation and semen deposition to trigger the ovulatory process (21), a close relationship has been observed between follicular size, plasma concentration of estradiol-17β, and LH secretion. Females with small follicles (<6 mm) secrete lower concentrations of estradiol-17β (12, 22) and respond to mating with less LH release (2). A low percentage of llamas (23) and camels (24) may ovulate spontaneously. In non-mated dromedary camels, it has been suggested that endogenous estradiol could induce GnRH release and subsequently stimulate preovulatory LH surge in a low percentage of female in specific occasions such as lactation or after a progesterone phase (24). Results hereby presented allow suggesting that a similar mechanism might be involved in llamas that ovulate spontaneously.

The pattern of plasma progesterone release after induction of ovulation with an injection of a GnRH analog in this study is similar to that previously reported in other studies in llamas. Plasma progesterone concentration starts to increase on Day 4 and reaches maximum concentration at Day 8, and a decrease is observed between Days 10 and 12 after induction of ovulation (25, 26).

The observation that females treated with 1.6 mg of estradiol-17β achieved plasma progesterone concentration above 1 ng ml^−1^ and the highest plasma progesterone concentration 1 day later than animals treated with the GnRH analog could be in relation to the time elapsed between treatment and the LH surge and therefore the moment of ovulation. Previous studies in llamas (27, 28) and cows (29) have reported that LH peak occurs ~2 h after injection of a GnRH analog. In addition, it has been shown in llamas that ovulation occurs around 29 h after treatment (7). Although there are no reports demonstrating the time elapsed between estradiol injection and LH surge in camelids, it could be speculated that the LH peak might occur between 12 and 18 h post injection, as previously reported in other species [cows: (30, 31); ewes: (8)]. Even though the time elapsed between treatment and ovulation was not evaluated in the present study, it could be suggested that llamas treated with estradiol-17β would have a retarded LH peak and consequently ovulate some hours later than those treated with a GnRH analog injection. Thereafter, it would determine a later development and functionality of the CL.

In summary, the results of the present study demonstrate that an injection of estradiol-17β is able to induce ovulation in llamas and that the response depends on the dose used. Most of the animals required the highest tested dose (1.6 mg) to induce the ovulatory process. Although the CL diameter in females induced to ovulate with estradiol was similar to that in llamas induced to ovulate with a GnRH analog, the rise in plasma progesterone concentration above 1 ng ml^−1^ and the peak progesterone concentration were attained 1 day later in the estradiol treated females. This information enables a better understanding of the ovulatory process in llamas, and the injection of estradiol-17β could be considered as a tool to develop new strategies to control ovarian activity in this species.

## Data Availability Statement

The raw data supporting the conclusions of this article will be made available by the authors, without undue reservation.

## Ethics Statement

The animal study was reviewed and approved by Faculty of Veterinary Sciences, UNCPBA.

## Author Contributions

CB Bianchi conducted the fieldwork, collected the data, performed the hormonal analysis, and interpreted the data and prepared the manuscript. MB and FV contributed with the fieldwork and collaborated with the preparation of the manuscript. MG contributed to analyzing the data, interpreting the results, and preparing the manuscript. MA contributed to the designing of the experiment, collecting data, interpreting the results, and critical reviewing of the manuscript. All authors contributed to the article and approved the submitted version.

## Conflict of Interest

The authors declare that the research was conducted in the absence of any commercial or financial relationships that could be construed as a potential conflict of interest.
